# Bis[*N*′-(2-methyl­propyl­idene)-4-(prop-2-en-1-yloxy)benzohydrazidato-κ^2^*N*′,*O*]nickel(II)

**DOI:** 10.1107/S2414314625009885

**Published:** 2025-11-11

**Authors:** Sultana Shakila Khan, Md. Belayet Hossain Howlader, Md. Chanmiya Sheikh, Ryuta Miyatake, Ennio Zangrando

**Affiliations:** a Department of Pharmacy, Pabna University of Science and Technology, Pabna-6600, Bangladesh; b Department of Chemistry, Rajshahi University, Rajshahi-6205, Bangladesh; c Division of Applied Chemistry, Graduate School of Natural Science and Technology, Okayama University, 1-1 Tsushima-naka, 3-Chome, Okayama, 700-8530, Japan; d Center for Environmental Conservation and Research Safety University of Toyama, 3190 Gofuku Toyama 930-8555 Japan; e Department of Chemical and Pharmaceutical Science, University of Trieste, Italy; Vienna University of Technology, Austria

**Keywords:** crystal structure, nickel(II) complex, aroylhydrazone ligand

## Abstract

In the mononuclear title complex, [Ni(C_14_H_17_N_2_O_2_)_2_], the nickel(II) atom exhibits a slightly distorted square-planar coordination environment with the metal located on a crystallographic center of symmetry that induces a *trans* configuration of the *N*,*O* chelating ligands. In the crystal, weak C—H⋯O and C—H⋯π inter­actions consolidate the packing.

## Abstract 2

In the bis-chelated mononuclear nickel(II) title complex with benzohydrazide ligands bearing an all­yloxy group, the nickel(II) atom exhibits a slightly distorted square-planar coordination environment with the metal located on a crystallographic center of symmetry that induces a *trans* configuration of the *N*,*O* chelating ligands..

## Graphical abstract

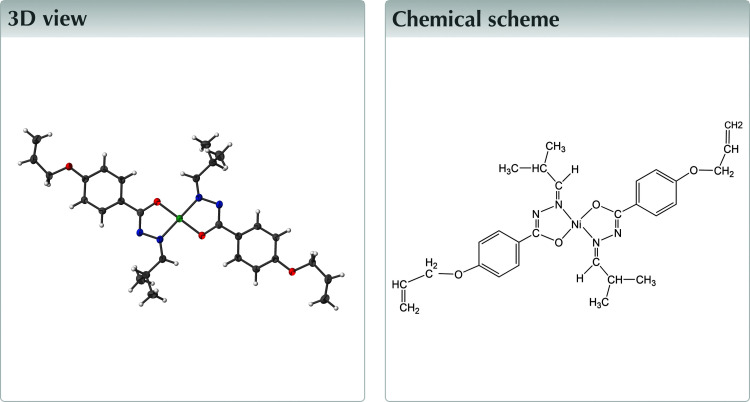

## Structure description

Hydrazone ligands have attracted special attention for their chelating capabilities. The corresponding nickel(II) complexes are of considerable inter­est since they exhibit a broad spectrum of structure-dependent physiological and pharmacological activities (Al-Qadsy *et al.*, 2021 ▸; Neethu *et al.*, 2021 ▸; Krishnamoorthy *et al.*, 2012 ▸; Yang *et al.*, 2020 ▸).

**Figure 1:**
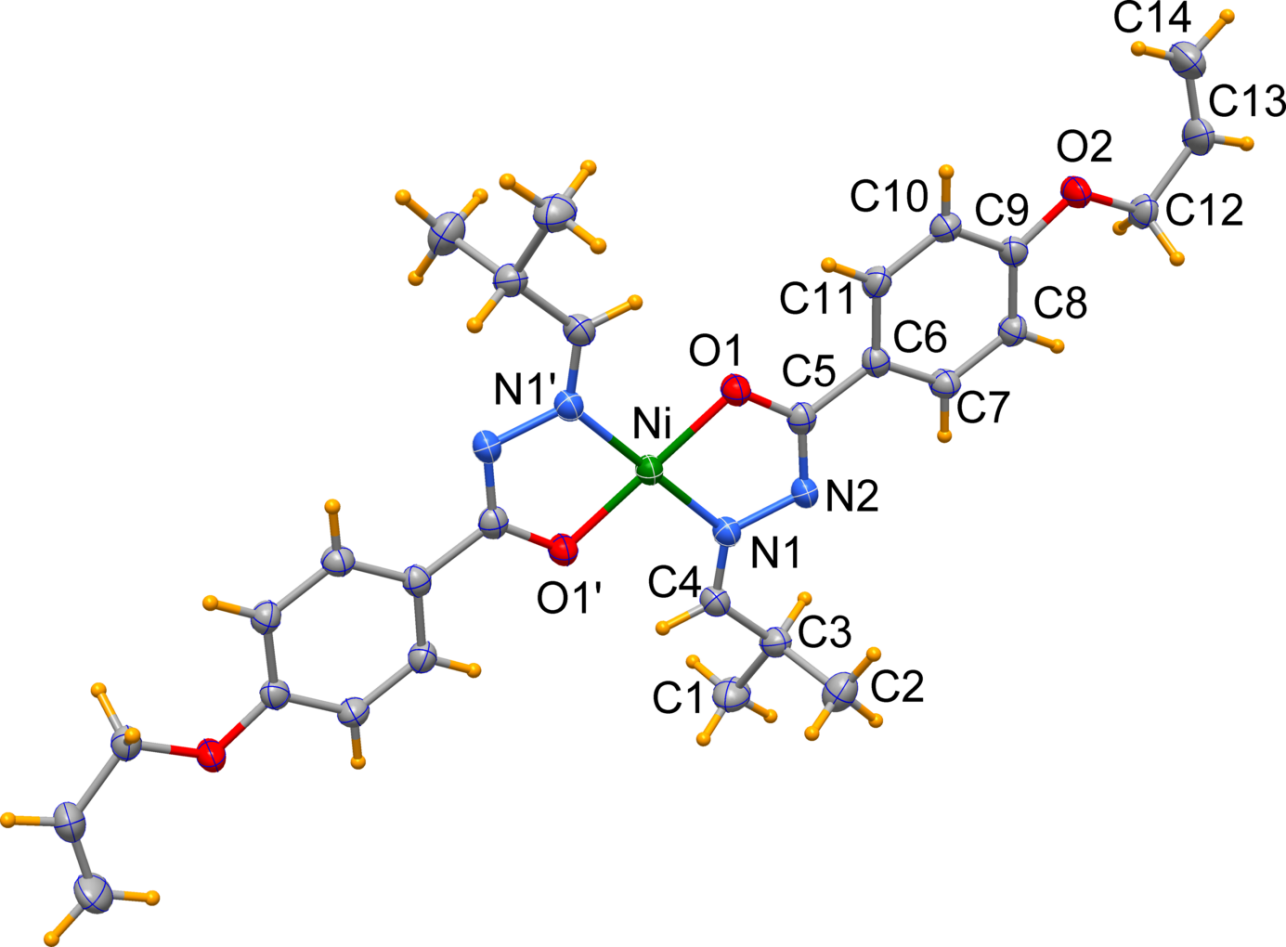
The mol­ecular structure of the title complex with displacement ellipsoids drawn at the 50% probability level. Atoms marked with a prime character and all non-labelled atoms are generated by inversion symmetry. [Symmetry code: −*x* + 1, −*y* + 1, −*z*.]

The title nickel(II) complex crystallizes in the monoclinic space group *P*2_1_/*c* with the metal located on an inversion center, so that the asymmetric unit comprises a half mol­ecule (Fig. 1 ▸). The Ni^II^ atom exhibits a slightly distorted square-planar coordination environment with the chelating ligands in a *trans* configuration imposed by symmetry. The coordinating enolizable O atom and the azomethine N atom of the deprotonated ligand form a five-membered nearly planar chelate ring (r.m.s. deviation from planarity = 0.0084 Å). The Ni—N1 and Ni—O1 bond lengths of 1.8671 (13) and 1.8382 (11) Å and the chelate angle of 83.64 (5)° are in agreement with those of previously reported complexes (Al-Qadsy *et al.*, 2021 ▸; Khan *et al.*, 2023 ▸, 2025 ▸; Krishnamoorthy *et al.*, 2012 ▸; Neethu *et al.*, 2021 ▸; Yang *et al.*, 2020 ▸), irrespective of the substituents present in the ligand. The C5—O1 bond length of 1.3080 (18) Å lies in between a C—O single and a C=O double bond. The bond lengths N1—C4 of 1.285 (2) Å and N2—C5 of 1.308 (2) Å are indicative of a conjugated system within the —CH=N—N=C—O fragment, even after the deprotonation of its enolized carbonyl O atom. The benzyl­idene entity (C5–C11) is practically co-planar with the N_2_O_2_ coordination plane [dihedral angle of 3.09 (5)°] and also to the all­yloxy fragment [7.73 (14)°]. An intra­molecular hydrogen bond between a methine group (C4—H4) and the chelating O atom (Table 1 ▸) stabilizes the mol­ecular conformation.

**Figure 2:**
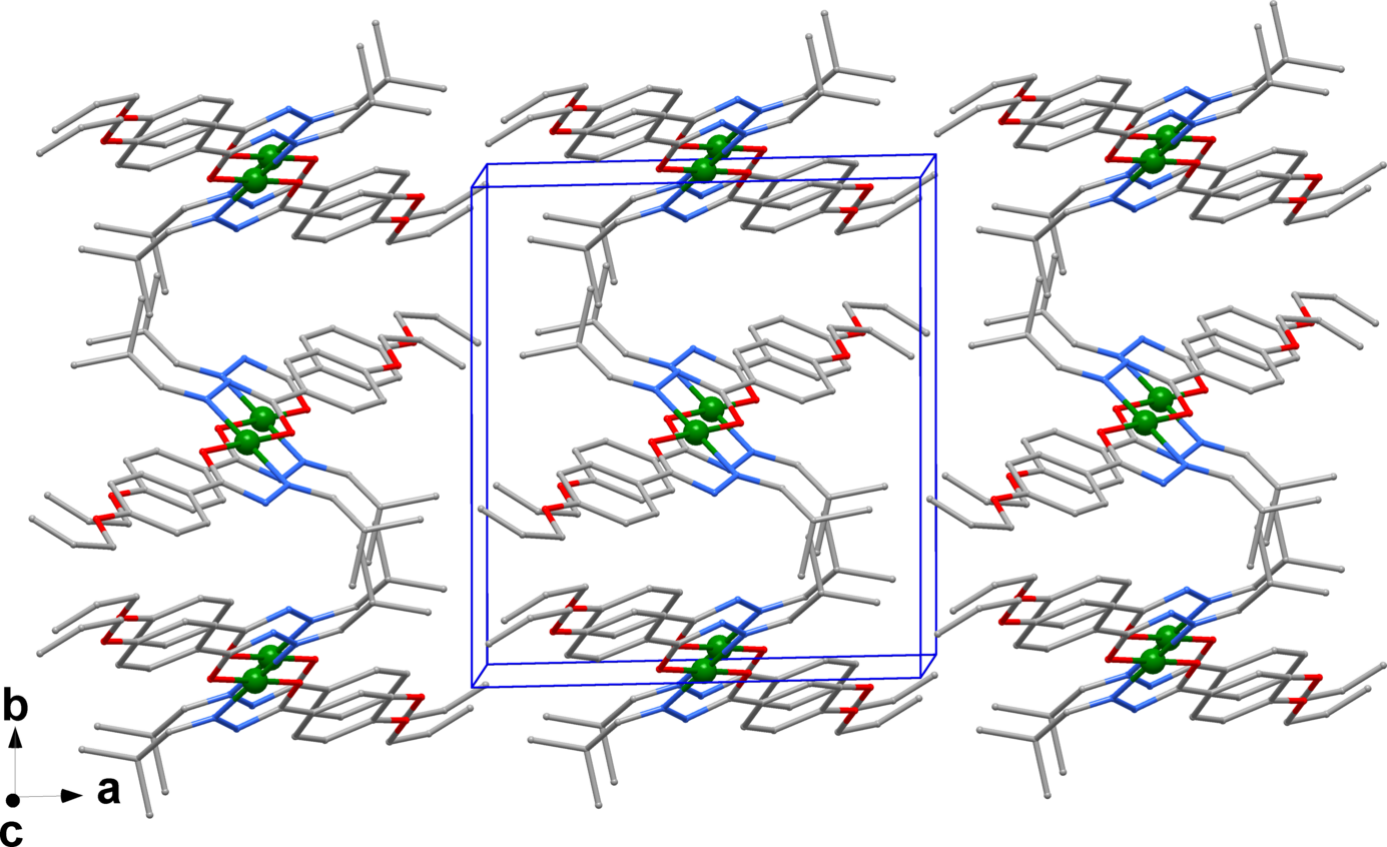
Crystal packing of the title complex with H atoms removed for clarity.

The packing of the complex mol­ecules is consolidated by a weak inter­molecular hydrogen bond between a methyl­ene group (C12—H12*A*) and the ligating O atom of a neighbouring complex. C—H⋯π inter­actions between the second H atom of this methyl­ene group and the centroid (*Cg*3) of the benzyl ring are also observed (Table 1 ▸), while no apparent π–π inter­actions are present. In the crystal packing (Fig. 2 ▸) the shortest separation of Ni^II^ atoms is 8.5593 (2) Å.

## Synthesis and crystallization

Isobutyraldehyde (0.216 g, 3.0 mmol) in 10 ml of ethanol was added to a 30 ml ethano­lic solution of 4-(all­yloxy)benzoyl­hydrazine (0.576 g, 3.0 mmol), followed by refluxing for one h. To this mixture Ni(CH_3_COO)_2_·4H_2_O (0.373 g, 1.5 mmol, in 30 ml) was introduced, and refluxing was prolonged for additional three h. The yellow precipitate formed was then filtered off while hot. Finally, the product was dried and stored in a vacuum desiccator containing anhydrous CaCl_2_. Single crystals of the nickel(II) complex, suitable for X-ray diffraction, were obtained through gradual evaporation from a mixture of chloro­form and toluene (3:1, *v*/*v*) over a period of 3 weeks. Yellow crystals, yield: 0.604 g (73%); melting point: 485–487 K. IR data (KBr disc, cm^−1^): 1606 ν(C=N), 1587 ν(C=C), 997 ν(N—N), 596 ν(*M*—N), 465 ν(*M*—O). ^1^H NMR (CDCl_3_, 400 MHz), δ: 7.82 (*d*, 2×2H, C-6, 8, *J* = 8.8 Hz), 6.85 (*d*, 2×2H, C-5, 9, *J* = 8.8 Hz), 6.50 (*d*, 2×1H, C-11, CH=N, *J* = 8.0 Hz), 6.4–6.0 (*m*, 2×1H, C-2, H_c_), 5.41 (*d*, 2×1H, C-1, H_a_, *J* = 17.6 Hz), 5.29 (*d*, 2×1H, C-1, H_b_, *J* = 10.4 Hz), 4.56 (*d*, 2×2H, C-3, OCH_2_, *J* = 5.6 Hz,), 3.72–3.63 (*m*, 2×1H, C-12), 1.16 (*d*, 2×6H, C-13,14, *J* = 8.4 Hz). HRMS (FAB) calculated for C_28_H_34_N_4_NiO_4_, [*M*+H]^+^: 549.20086, found [*M*+H]^+^: 549.20062.

## Refinement

Crystal data, data collection, and structure refinement details are summarized in Table 2 ▸.

## Supplementary Material

Crystal structure: contains datablock(s) I, global. DOI: 10.1107/S2414314625009885/wm4238sup1.cif

Structure factors: contains datablock(s) I. DOI: 10.1107/S2414314625009885/wm4238Isup2.hkl

CCDC reference: 2335124

Additional supporting information:  crystallographic information; 3D view; checkCIF report

## Figures and Tables

**Table 1:** Hydrogen-bond geometry (Å, °) *Cg*3 is the centroid of the benzyl ring.

*D*—H⋯*A*	*D*—H	H⋯*A*	*D*⋯*A*	*D*—H⋯*A*
C4—H4⋯O1^i^	0.95	2.47	2.9822 (19)	114
C12—H12*A*⋯O1^ii^	0.99	2.58	3.451 (2)	148
C12—H12*B*⋯*Cg*3^iii^	0.99	2.64	3.4762 (17)	143

Symmetry codes: (i) 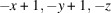; (ii) 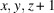; (iii) 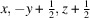.

**Table 2:** Experimental details

Crystal data	
Chemical formula	[Ni(C_14_H_17_N_2_O_2_)_2_]
*M*_r_	549.30
Crystal system, space group	Monoclinic, *P*2_1_/*c*
Temperature (K)	173
*a*, *b*, *c* (Å)	12.5241 (3), 13.4196 (4), 8.5593 (2)
β (°)	109.274 (8)
*V* (Å^3^)	1357.91 (9)
*Z*	2
Radiation type	Mo *K*α
μ (mm^−1^)	0.75
Crystal size (mm)	0.22 × 0.11 × 0.07

Data collection	
Diffractometer	Rigaku R-AXIS RAPID
Absorption correction	Multi-scan (*ABSCOR*; Rigaku, 1995 ▸)
*T*_min_, *T*_max_	0.722, 0.949
No. of measured, independent and observed [*I* > 2σ(*I*)] reflections	12824, 3106, 2678
*R*_int_	0.031
(sin θ/λ)_max_ (Å^−1^)	0.649

Refinement	
*R*[*F*^2^ > 2σ(*F*^2^)], *wR*(*F*^2^), *S*	0.032, 0.081, 1.04
No. of reflections	3106
No. of parameters	171
H-atom treatment	H-atom parameters constrained
Δρ_max_, Δρ_min_ (e Å^−3^)	0.43, −0.22

Computer programs: *RAPID-AUTO* (Rigaku, 2019 ▸), *SHELXT* (Sheldrick, 2015*a* ▸), *SHELXL* (Sheldrick, 2015*b* ▸), *DIAMOND* (Brandenburg & Putz, 1999 ▸) and *WinGX* (Farrugia, 2012 ▸).

## full crystallographic data

### Bis[N'-(2-methylpropylidene)-4-(prop-2-en-1-yloxy)benzohydrazidato-κ2N',O]nickel(II) Crystal data

**Table:**

[Ni(C_14_H_17_N_2_O_2_)_2_]	*F*(000) = 580
*M**_r_* = 549.30	*D*_x_ = 1.343 Mg m^−^^3^
Monoclinic, *P*2_1_/*c*	Mo *K*α radiation, λ = 0.71075 Å
*a* = 12.5241 (3) Å	Cell parameters from 10492 reflections
*b* = 13.4196 (4) Å	θ = 2.3–27.5°
*c* = 8.5593 (2) Å	µ = 0.75 mm^−^^1^
β = 109.274 (8)°	*T* = 173 K
*V* = 1357.91 (9) Å^3^	Prism, yellow
*Z* = 2	0.22 × 0.11 × 0.07 mm

### Bis[N'-(2-methylpropylidene)-4-(prop-2-en-1-yloxy)benzohydrazidato-κ2N',O]nickel(II) Data collection

**Table:**

Rigaku R-AXIS RAPID diffractometer	2678 reflections with *I* > 2σ(*I*)
ω scans	*R*_int_ = 0.031
Absorption correction: multi-scan (*ABSCOR*; Rigaku, 1995)	θ_max_ = 27.5°, θ_min_ = 2.3°
*T*_min_ = 0.722, *T*_max_ = 0.949	*h* = −16→16
12824 measured reflections	*k* = −17→17
3106 independent reflections	*l* = −11→11

### Bis[N'-(2-methylpropylidene)-4-(prop-2-en-1-yloxy)benzohydrazidato-κ2N',O]nickel(II) Refinement

**Table:**

Refinement on *F*^2^	0 restraints
Least-squares matrix: full	Hydrogen site location: inferred from neighbouring sites
*R*[*F*^2^ > 2σ(*F*^2^)] = 0.032	H-atom parameters constrained
*wR*(*F*^2^) = 0.081	*w* = 1/[σ^2^(*F*_o_^2^) + (0.0388*P*)^2^ + 0.5845*P*] where *P* = (*F*_o_^2^ + 2*F*_c_^2^)/3
*S* = 1.04	(Δ/σ)_max_ < 0.001
3106 reflections	Δρ_max_ = 0.43 e Å^−^^3^
171 parameters	Δρ_min_ = −0.21 e Å^−^^3^

### Bis[N'-(2-methylpropylidene)-4-(prop-2-en-1-yloxy)benzohydrazidato-κ2N',O]nickel(II) Special details

**Table:**

Geometry. All esds (except the esd in the dihedral angle between two l.s. planes) are estimated using the full covariance matrix. The cell esds are taken into account individually in the estimation of esds in distances, angles and torsion angles; correlations between esds in cell parameters are only used when they are defined by crystal symmetry. An approximate (isotropic) treatment of cell esds is used for estimating esds involving l.s. planes.

### Bis[N'-(2-methylpropylidene)-4-(prop-2-en-1-yloxy)benzohydrazidato-κ2N',O]nickel(II) Fractional atomic coordinates and isotropic or equivalent isotropic displacement parameters (Å^2^)

**Table:**

	*x*	*y*	*z*	*U*_iso_*/*U*_eq_	
Ni1	0.500000	0.500000	0.000000	0.02365 (9)	
O1	0.40545 (9)	0.48867 (8)	0.12389 (13)	0.0272 (2)	
O2	0.18732 (9)	0.38385 (9)	0.66963 (13)	0.0302 (3)	
N1	0.59615 (11)	0.42033 (10)	0.16621 (15)	0.0251 (3)	
N2	0.55099 (11)	0.39157 (10)	0.28959 (16)	0.0265 (3)	
C1	0.89710 (15)	0.35433 (15)	0.3488 (3)	0.0423 (4)	
H1A	0.913977	0.331595	0.250634	0.051*	
H1B	0.945166	0.318522	0.446480	0.051*	
H1C	0.911834	0.426013	0.363773	0.051*	
C2	0.74500 (17)	0.22294 (14)	0.3009 (2)	0.0411 (4)	
H2A	0.798169	0.184695	0.390729	0.049*	
H2B	0.750983	0.201381	0.194659	0.049*	
H2C	0.667728	0.211635	0.300996	0.049*	
C3	0.77329 (14)	0.33389 (12)	0.3261 (2)	0.0290 (3)	
H3	0.758739	0.355730	0.428933	0.035*	
C4	0.69787 (14)	0.39313 (12)	0.18425 (19)	0.0274 (3)	
H4	0.727860	0.412281	0.100237	0.033*	
C5	0.45128 (13)	0.43305 (11)	0.25439 (18)	0.0245 (3)	
C6	0.38466 (13)	0.41678 (11)	0.36654 (18)	0.0239 (3)	
C7	0.42822 (13)	0.36377 (12)	0.51408 (19)	0.0264 (3)	
H7	0.502209	0.336480	0.543159	0.032*	
C8	0.36562 (13)	0.35007 (12)	0.61932 (19)	0.0268 (3)	
H8	0.396352	0.313752	0.719449	0.032*	
C9	0.25697 (13)	0.39030 (11)	0.57628 (18)	0.0247 (3)	
C10	0.21195 (14)	0.44225 (12)	0.42783 (19)	0.0278 (3)	
H10	0.137429	0.468481	0.397653	0.033*	
C11	0.27520 (13)	0.45567 (12)	0.32482 (19)	0.0264 (3)	
H11	0.244144	0.491699	0.224447	0.032*	
C12	0.22916 (14)	0.33458 (12)	0.82672 (19)	0.0289 (3)	
H12A	0.301977	0.364646	0.894171	0.035*	
H12B	0.242193	0.263153	0.810353	0.035*	
C13	0.14474 (17)	0.34507 (15)	0.9136 (2)	0.0416 (4)	
H13	0.160758	0.310432	1.015534	0.050*	
C14	0.05119 (18)	0.39645 (17)	0.8655 (3)	0.0511 (5)	
H14A	0.030867	0.432590	0.764502	0.061*	
H14B	0.003345	0.397763	0.931571	0.061*	

### Bis[N'-(2-methylpropylidene)-4-(prop-2-en-1-yloxy)benzohydrazidato-κ2N',O]nickel(II) Atomic displacement parameters (Å^2^)

**Table:**

	*U*^11^	*U*^22^	*U*^33^	*U*^12^	*U*^13^	*U*^23^
Ni1	0.02442 (15)	0.02543 (15)	0.02219 (15)	0.00068 (11)	0.00916 (10)	0.00268 (11)
O1	0.0265 (5)	0.0313 (6)	0.0251 (5)	0.0021 (5)	0.0102 (4)	0.0048 (4)
O2	0.0293 (6)	0.0368 (6)	0.0278 (6)	0.0051 (5)	0.0139 (5)	0.0085 (5)
N1	0.0277 (6)	0.0259 (6)	0.0237 (6)	0.0000 (5)	0.0111 (5)	0.0009 (5)
N2	0.0291 (7)	0.0281 (7)	0.0253 (6)	0.0002 (5)	0.0130 (5)	0.0033 (5)
C1	0.0313 (9)	0.0417 (10)	0.0533 (11)	0.0065 (8)	0.0132 (8)	0.0076 (9)
C2	0.0491 (11)	0.0308 (9)	0.0409 (10)	0.0014 (8)	0.0114 (8)	0.0049 (8)
C3	0.0296 (8)	0.0293 (8)	0.0288 (8)	0.0042 (7)	0.0107 (6)	0.0013 (6)
C4	0.0301 (8)	0.0282 (8)	0.0263 (7)	0.0005 (6)	0.0127 (6)	0.0008 (6)
C5	0.0275 (7)	0.0226 (7)	0.0234 (7)	−0.0035 (6)	0.0086 (6)	−0.0016 (6)
C6	0.0251 (7)	0.0233 (7)	0.0240 (7)	−0.0029 (6)	0.0091 (6)	−0.0001 (6)
C7	0.0232 (7)	0.0290 (8)	0.0268 (7)	0.0017 (6)	0.0079 (6)	0.0013 (6)
C8	0.0286 (8)	0.0271 (8)	0.0243 (7)	0.0016 (6)	0.0083 (6)	0.0044 (6)
C9	0.0271 (8)	0.0229 (7)	0.0260 (7)	−0.0015 (6)	0.0114 (6)	−0.0002 (6)
C10	0.0265 (8)	0.0281 (8)	0.0292 (8)	0.0047 (6)	0.0099 (6)	0.0042 (6)
C11	0.0299 (8)	0.0242 (7)	0.0251 (7)	0.0020 (6)	0.0088 (6)	0.0042 (6)
C12	0.0347 (9)	0.0279 (8)	0.0251 (7)	0.0009 (7)	0.0111 (6)	0.0040 (6)
C13	0.0523 (11)	0.0428 (10)	0.0383 (10)	0.0070 (9)	0.0265 (9)	0.0117 (8)
C14	0.0483 (12)	0.0591 (13)	0.0584 (13)	0.0099 (10)	0.0345 (10)	0.0159 (10)

### Bis[N'-(2-methylpropylidene)-4-(prop-2-en-1-yloxy)benzohydrazidato-κ2N',O]nickel(II) Geometric parameters (Å, º)

**Table:**

Ni1—O1^i^	1.8382 (11)	C4—H4	0.9500
Ni1—O1	1.8382 (11)	C5—C6	1.481 (2)
Ni1—N1^i^	1.8671 (13)	C6—C7	1.394 (2)
Ni1—N1	1.8671 (13)	C6—C11	1.399 (2)
O1—C5	1.3080 (18)	C7—C8	1.388 (2)
O2—C9	1.3663 (18)	C7—H7	0.9500
O2—C12	1.4334 (18)	C8—C9	1.396 (2)
N1—C4	1.285 (2)	C8—H8	0.9500
N1—N2	1.4065 (17)	C9—C10	1.395 (2)
N2—C5	1.308 (2)	C10—C11	1.378 (2)
C1—C3	1.523 (2)	C10—H10	0.9500
C1—H1A	0.9800	C11—H11	0.9500
C1—H1B	0.9800	C12—C13	1.486 (2)
C1—H1C	0.9800	C12—H12A	0.9900
C2—C3	1.529 (2)	C12—H12B	0.9900
C2—H2A	0.9800	C13—C14	1.304 (3)
C2—H2B	0.9800	C13—H13	0.9500
C2—H2C	0.9800	C14—H14A	0.9500
C3—C4	1.497 (2)	C14—H14B	0.9500
C3—H3	1.0000		
			
O1^i^—Ni1—O1	180.0	O1—C5—N2	123.77 (14)
O1^i^—Ni1—N1^i^	83.64 (5)	O1—C5—C6	117.08 (13)
O1—Ni1—N1^i^	96.36 (5)	N2—C5—C6	119.15 (13)
O1^i^—Ni1—N1	96.36 (5)	C7—C6—C11	118.63 (14)
O1—Ni1—N1	83.64 (5)	C7—C6—C5	121.78 (14)
N1^i^—Ni1—N1	180.0	C11—C6—C5	119.59 (14)
C5—O1—Ni1	110.80 (10)	C8—C7—C6	121.30 (14)
C9—O2—C12	118.13 (12)	C8—C7—H7	119.4
C4—N1—N2	117.41 (13)	C6—C7—H7	119.4
C4—N1—Ni1	128.23 (11)	C7—C8—C9	119.21 (14)
N2—N1—Ni1	114.28 (10)	C7—C8—H8	120.4
C5—N2—N1	107.47 (12)	C9—C8—H8	120.4
C3—C1—H1A	109.5	O2—C9—C10	114.85 (13)
C3—C1—H1B	109.5	O2—C9—C8	125.18 (13)
H1A—C1—H1B	109.5	C10—C9—C8	119.97 (14)
C3—C1—H1C	109.5	C11—C10—C9	120.22 (14)
H1A—C1—H1C	109.5	C11—C10—H10	119.9
H1B—C1—H1C	109.5	C9—C10—H10	119.9
C3—C2—H2A	109.5	C10—C11—C6	120.66 (14)
C3—C2—H2B	109.5	C10—C11—H11	119.7
H2A—C2—H2B	109.5	C6—C11—H11	119.7
C3—C2—H2C	109.5	O2—C12—C13	108.98 (13)
H2A—C2—H2C	109.5	O2—C12—H12A	109.9
H2B—C2—H2C	109.5	C13—C12—H12A	109.9
C4—C3—C1	110.55 (14)	O2—C12—H12B	109.9
C4—C3—C2	110.35 (14)	C13—C12—H12B	109.9
C1—C3—C2	111.84 (15)	H12A—C12—H12B	108.3
C4—C3—H3	108.0	C14—C13—C12	127.31 (17)
C1—C3—H3	108.0	C14—C13—H13	116.3
C2—C3—H3	108.0	C12—C13—H13	116.3
N1—C4—C3	125.70 (14)	C13—C14—H14A	120.0
N1—C4—H4	117.1	C13—C14—H14B	120.0
C3—C4—H4	117.1	H14A—C14—H14B	120.0
			
N1^i^—Ni1—O1—C5	179.15 (10)	N2—C5—C6—C7	4.2 (2)
N1—Ni1—O1—C5	−0.84 (10)	O1—C5—C6—C11	3.6 (2)
O1^i^—Ni1—N1—C4	4.90 (15)	N2—C5—C6—C11	−176.09 (14)
O1—Ni1—N1—C4	−175.10 (15)	C11—C6—C7—C8	−0.6 (2)
O1^i^—Ni1—N1—N2	−178.37 (10)	C5—C6—C7—C8	179.14 (14)
O1—Ni1—N1—N2	1.63 (10)	C6—C7—C8—C9	0.0 (2)
C4—N1—N2—C5	175.12 (14)	C12—O2—C9—C10	−177.85 (14)
Ni1—N1—N2—C5	−1.98 (15)	C12—O2—C9—C8	1.9 (2)
N2—N1—C4—C3	−0.5 (2)	C7—C8—C9—O2	−178.80 (14)
Ni1—N1—C4—C3	176.19 (12)	C7—C8—C9—C10	0.9 (2)
C1—C3—C4—N1	−154.63 (17)	O2—C9—C10—C11	178.54 (14)
C2—C3—C4—N1	81.1 (2)	C8—C9—C10—C11	−1.2 (2)
Ni1—O1—C5—N2	−0.14 (19)	C9—C10—C11—C6	0.6 (2)
Ni1—O1—C5—C6	−179.85 (10)	C7—C6—C11—C10	0.3 (2)
N1—N2—C5—O1	1.4 (2)	C5—C6—C11—C10	−179.43 (14)
N1—N2—C5—C6	−178.90 (12)	C9—O2—C12—C13	174.40 (14)
O1—C5—C6—C7	−176.10 (14)	O2—C12—C13—C14	−5.0 (3)

Symmetry code: (i) −*x*+1, −*y*+1, −*z*.

### Bis[N'-(2-methylpropylidene)-4-(prop-2-en-1-yloxy)benzohydrazidato-κ2N',O]nickel(II) Hydrogen-bond geometry (Å, º)

*Cg*3 is the centroid of the benzyl ring.

**Table:**

*D*—H···*A*	*D*—H	H···*A*	*D*···*A*	*D*—H···*A*
C4—H4···O1^i^	0.95	2.47	2.9822 (19)	114
C12—H12*A*···O1^ii^	0.99	2.58	3.451 (2)	148
C12—H12*B*···*Cg*3^iii^	0.99	2.64	3.4762 (17)	143

Symmetry codes: (i) −*x*+1, −*y*+1, −*z*; (ii) *x*, *y*, *z*+1; (iii) *x*, −*y*+1/2, *z*+1/2.
